# Smart Vehicle Path Planning Based on Modified PRM Algorithm

**DOI:** 10.3390/s22176581

**Published:** 2022-08-31

**Authors:** Qiongqiong Li, Yiqi Xu, Shengqiang Bu, Jiafu Yang

**Affiliations:** College of Mechanical and Electronic Engineering, Nanjing Forestry University, Nanjing 210037, China

**Keywords:** smart vehicle, probabilistic roadmap algorithm, pseudo-random sampling, collision detection, path smoothing

## Abstract

Path planning is a very important step for mobile smart vehicles in complex environments. Sampling based planners such as the Probabilistic Roadmap Method (PRM) have been widely used for smart vehicle applications. However, there exist some shortcomings, such as low efficiency, low reuse rate of the roadmap, and a lack of guidance in the selection of sampling points. To solve the above problems, we designed a pseudo-random sampling strategy with the main spatial axis as the reference axis. We optimized the generation of sampling points, removed redundant sampling points, set the distance threshold between road points, adopted a two-way incremental method for collision detections, and optimized the number of collision detection calls to improve the construction efficiency of the roadmap. The key road points of the planned path were extracted as discrete control points of the Bessel curve, and the paths were smoothed to make the generated paths more consistent with the driving conditions of vehicles. The correctness of the modified PRM was verified and analyzed using MATLAB and ROS to build a test platform. Compared with the basic PRM algorithm, the modified PRM algorithm has advantages related to speed in constructing the roadmap, path planning, and path length.

## 1. Introduction

In recent years, smart vehicles have received more attention with the development of emerging technologies such as cloud computing, big data, and the full-scale launch of 5G construction [1,2]. Smart vehicles have significant effects in relieving driving pressure, avoiding traffic jams, and reducing environmental pollution [3] Path planning and motion control are significant and complex navigation tasks in smart vehicles. Path planning technology is the basis of smart vehicles to make motion decisions and navigate positioning [4,5]. To achieve successful path planning and motion control to be able to reach a target safely, smart vehicles must be provided with the ability to perceive and detect obstacles to be avoided [6]. Many sensors are installed on the body of smart vehicles, which ensure that they can perceive and interpret information gathered from the environment to determine position, direction to the target, position of obstacles, and navigation in both structured or unstructured environments [7].A smart vehicle is expected to perform these tasks with the safest and shortest path, reaching the target in the shortest time, and ultimately performing the specified task without the intervention of humans. Path planning in smart vehicles refers to determining how the smart vehicle reaches its target point safely to ensure obstacle avoidance. Smart vehicle path planning is described as a multi-objective optimization problem as it requires the generation of appropriate trajectories as well as obstacle avoidance in the environment [8].

The methods of smart vehicle path planning can be classified in different ways. Ayawli et al. [7] categorized them into nature-inspired computation methods, traditional methods, and hybrid methods. Methods and strategies that imitate natural phenomena are described as nature-inspired computation methods. Meanwhile those that have nothing to do with imitating nature phenomena are described as the conventional method. Approaches that combine two or more strategies are described as hybrid methods. Nature-inspired computing consists of a metaheuristic algorithm that simulates, based on nature phenomena given by natural science [9]. A number of researchers have attempted to solve the problem of mobile robotics path planning by using nature-inspired algorithms including genetic algorithms (GA) [10,11], artificial neural networks (ANN) [12,13], simulated annealing (SA) [14], ant colony optimization (ACO) [15], particle swarm optimization (PSO) [16], and artificial bee colonies (ABC) [17].In order to take advantage of the strengths of some methods while reducing the effects of their disadvantages, some researchers combine two or more methods to provide an efficient hybrid path planning method for controlling smart vehicles. These approaches include APF combined with GA [18], APF combined with PSO [19], and fuzzy logic combined with Kalman filtering [20,21]. Conventional path planning methods have been used for many years. These methods mainly rely on distance information from the object to the smart vehicles, repulsive force and attractive force clustering, or graphical map calculations to determine the path planning of smart vehicles. Even though conventional methods of path planning are computationally expensive, they are easy to implement. Conventional methods mainly consist of the rapidly-exploring random tree (RRT) algorithm [22], probabilistic roadmap algorithm (PRM) [23], artificial potential field (APF) [24,25], sliding mode control (SMC) [11,26], A * algorithm [27], D * algorithm [28,29], and simultaneous localization and mapping (SLAM) [30].

PRM is one of the most popular sampling based planners. PRM is a space planner that uses multiple-query planning. The key idea in PRM is to distribute the nodes across the space and then connect these nodes using simple local planning and straight lines to form a graph roadmap. By connecting the available space, the PRM succeeds in exploring a faster path by reducing the search to a graph [31]. However, PRM has shortcomings, including lack of orientation in the selection of sampling points, low reuse rate of the roadmap, and low search efficiency. Moreover, due to the random sampling of nodes in PRM, there exists a narrow passage problem that generates an unconnected graph. To enhance the efficiency of sampling-based algorithms, Kantaros et al. [32] introduced bias into the sampling process. Vasile et al. [33] maintained sparsity of generated samples. Sparseness was also explored by Dobson and Berkis for PRM using different techniques [34]. Amato et al. [35] proposed parallelizing strategies; the PRM method has massive inherent parallelism, which can be easily and best exploited. Berkis et al. [36] used the probabilistic roadmap method (PRM) with bidirectional rapidly exploring random trees (BI-RRT) as the local planner to solve multiple queries for motion planning problems with single query planners. Kurniawait et al. [37] designed an improved PRM algorithm, which was based on obstacle boundary sampling and evaluated the optimal feasible region to optimize the dispersion of random sampling of the PRM algorithm. Esposito et al. [38] proposed a processing algorithm for optimizing probabilistic roadmaps. Dealing with the format of convex cells in free space with a number of nodes that requires a lot of computation, this algorithm could simplify the computation required for this step by sparse decomposition. Gao Junli et al. [39] proposed to combine the deep reinforcement learning twin-delayed deep deterministic policy gradient algorithm with the traditional PRM algorithm as a new path planner, and the experimental results showed that this incremental training mode could significantly improve search efficiency. Moreover, this new path planner effectively improved the generalization of the model. Chen Gang et al. [40] proposed an improved PRM method. Based on a virtual force field, a new sampling strategy of PRM was proposed to generate a configuration that is more appropriate for practical application in free space.

RAVANKAR et al. [41] proposed a method for global planning using a hierarchical hybrid PRM and the APF method, using a decomposition method of node distribution that used map segmentation to generate regions of high and low potential, and proposed a method to reduce the dispersion of sample sets during roadmap building. Xu Zhenfan et al. [42] changed the sampling strategy so that nodes were incrementally added and evenly distributed in the exploration region to produce the best viewpoints and PRM enabled the planner to quickly search for alternative paths and avoid dynamic obstacles for safe exploration.

Aiming to improve the shortcomings of the PRM algorithm, the main innovation of this paper is that we propose a pseudo-random sampling strategy with the main spatial axis as the reference axis, set the distance threshold between road points, and adopt a two-way incremental method for collision detections. We aim to find the shortest path between the start point and target point and shorten the time of the planning path. The key road points of the path are extracted as discrete control points of the Bessel curve. We use Bezier curve to make the path smoother, whereas the path is more like the actual driving condition of the smart vehicle.

## 2. Modified PRM Algorithm

### 2.1. PRM Algorithm

The PRM algorithm includes sampling and query phases.

Sampling phase: the PRM algorithm randomly samples in the planning space and judges the reasonableness of the sampling points by the local planner. By repeating the sampling times n to generate a collection of valid waypoints V traversing the V, the algorithm connects all the feasible paths between the waypoints to expand to the whole planning space and forms the waypoint graph. $V=\{v_{1},.v_{2},\ldots ,v_{n}\}$ denotes the set of waypoints; $E=\{v_{i},v_{j}|v_{i},v_{j}\in V\}$ denotes the set of edges between waypoints.

Query phase: the start point $q_{init}$ and target point $q_{goal}$ are put into the wayfinding graph G(V,E), and the algorithm enters the path search phase. We use the graph search algorithm in the wayfinding graph G(V,E) to find a collision-free path connecting the start point $q_{init}$ and target point $q_{goal}$.

### 2.2. Pseudo-Random Sampling

In the PRM algorithm, the number of sampling points generated by the random sampling strategy increases with an increase in planning space. It is difficult to achieve a global uniform distribution and easy to create redundancy in sampling points. There is a considerable probability that the shortest path occurs in the area where the starting point and target point connects. This region is regarded as a focused sampling region, referred to as the spatial principal axis region.

To construct the spatial principal axis information, we set the coordinates of the starting point to be $S\left(x_{s},y_{s}\right)$ and the coordinates of the target point to be $\;G\left(x_{g},y_{g}\right)$. Length L and declination of the spatial principal axis θ was denoted by:

(1) $$L={\left\|G-S\right\|}_{2}$$

(2) $$\theta =\frac{\pi }{2}-\arctan\frac{\left|y_{g}-y_{s}\right|}{\left|x_{g}-x_{s}\right|}$$

We designed the spatial principal axes with the length L, and number of sampling points n, then obtained the longitudinal sampling spacing $N_{d}$, as:

(3) $$N_{d}=\frac{L}{n}$$

Referring to the random sampling method, the sampling points were symmetrically distributed in the sector area near the main axis of space, and sampling points $P_{i,j}(x,y)$ were calculated as follows:

(4) $$x=x_{s}+r_{d}\times \cos(\theta +\phi_{j})$$

(5) $$y=y_{s}+r_{d}\times \sin(\theta +\phi_{j})$$

(6) $$r_{d}=i\times N_{d},i=[1,2,\ldots ,n]$$

where $\left(x_{s},y_{s}\right)$ indicates the starting point of the intelligent vehicle; $r_{d}$ indicates the sampling radius; sampling radius is centered on the starting point; $\phi_{j}\in \left[-\phi_{m},\phi_{m}\right]$ indicates the angle of deflection of the sampling point and; $\phi_{m}$ indicates the maximum deflection angle. It is used to control the angle of the sector sampling area, that is, the range of lateral sampling.

**Figure 1:**
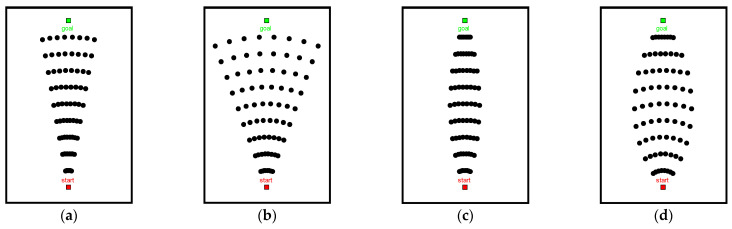
Sampling method based on spatial principal axis: (**a**) $\phi_{m}=10$, (**b**) $\phi_{m}=20$, (**c**) $\phi_{m}=10$, and (**d**) $\phi_{m}=20$.

According to Figure 1a,b, the sampling points are symmetrically distributed on both sides of the main spatial axis, and sampling range is controlled by the maximum deflection angle $\phi_{m}$. With the increase of $\phi_{m}$, the sampling points spread in all directions along the main spatial axis. To make the sampling point distribution more uniform, the lateral sampling range is adjusted along the main axis of space, and sampling range is adjusted in increments using $\Delta \phi =\phi_{m}/n$. The distribution of sampling points after adjustment is shown in Figure 1c,d.

Integrating the characteristics of uniform sampling, we counted the number of sampling points p in free space and the effective sampling rate of the horizontal sampling layer is defined as R:

(7) $$R=\frac{p}{N}$$

where N indicates the total number of samples in the current sampling layer and the size of the effective sampling rate R reflects the connectivity of the current sampling layer. The larger R is, the better the connectivity of the sampling layer. If R is too small, this means that most of the sampling points in the sampling layer have fallen into the obstacle space. If the sampling layer edge subsequently has the same sampling interval, the chance of sampling points falling into the obstacle space will increase.

**Figure 2:**
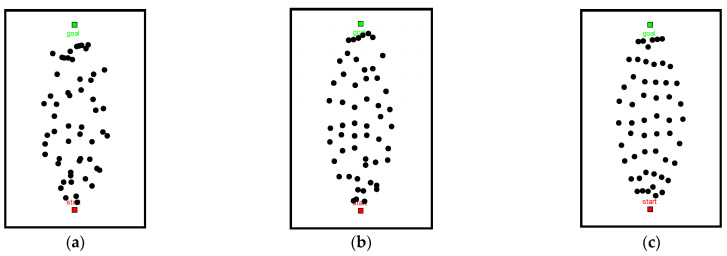
Pseudo-random-based sampling method: (**a**) Δr=r, (**b**) Δr=0.5r, and (**c**) Δr=0.25r.

In order to improve the ability of the sampling points in avoiding obstacles, we introduced random increments Δr to adjust the sampling interval of sampling points. Based on Figure 1d, we adjust the size of the random increment Δr to get Figure 2. As the value of the random increment Δr increases, the sampling points tend to approach random distribution. With a decreasing value of Δr, the sampling points tend to approach uniform distribution.

The sampling radius after adding random increments Δr is shown in Equation (8):

(8) $$r_{d}^{\prime }=r_{d}+\Delta r$$

**Figure 3:**
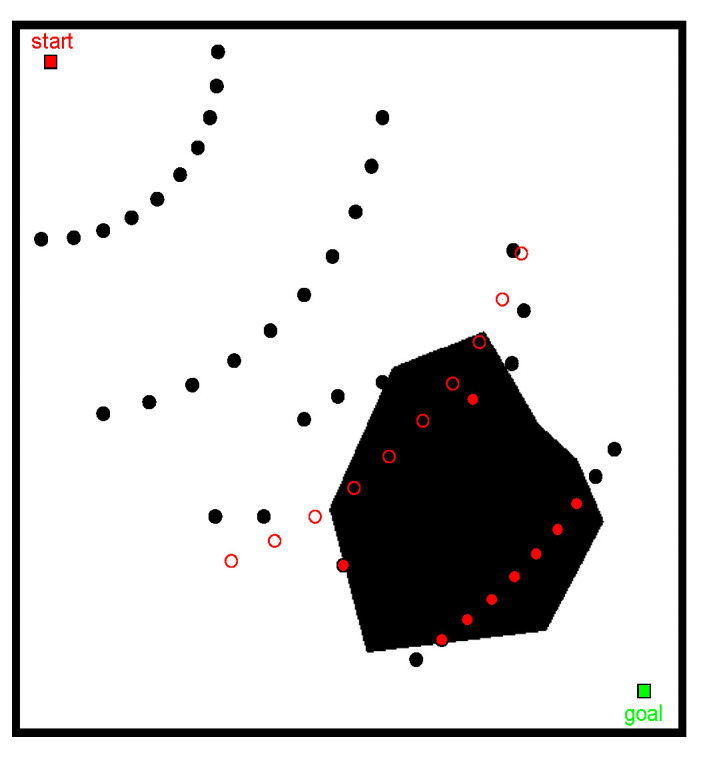
Schematic of sampling point adjustment.

Referring to Figure 3, hollow dots indicate the sampling points before adjusting the sampling spacing, solid dots indicate the adjusted sampling points, red markers represent the sampling points falling into the obstacle space, and black markers represent the sampling points in the free space. The effective sampling rate of the front sampling layer is low (R=0.3), the radius fluctuation rate (R=0.8) of the subsequent sampling layer is adjusted, and the sampling points avoid the obstacles by using the pseudo-random sampling strategy, which improves the quality of sampling point generation.

### 2.3. Bidirectional Incremental Collision Detection

Collision detection is used to determine whether the connected line segments between the sample points intersect with the obstacle space, and the sample points are connected to each other by collision detection to form a roadmap G(V,E). The traditional PRM algorithm usually takes an incremental detection strategy. According to a fixed step size, the planner selects discrete points and detects whether the point falls into the obstacle space. To improve the efficiency of collision detection execution, we combined this incremental detection method with the dichotomous method, proposing a two-way incremental detection strategy.

**Figure 4:**
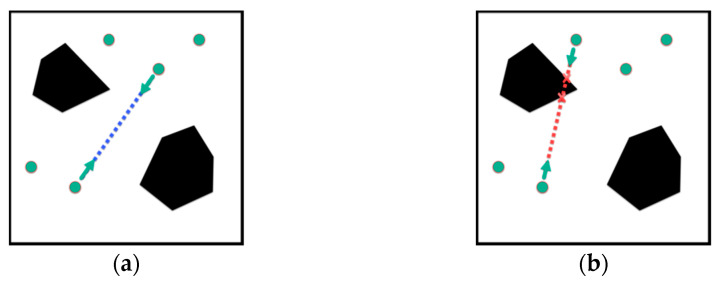
Schematic diagram of two-way incremental detection strategy: (**a**) reasonable path and (**b**) illegal path.

First, the two-way incremental detection method judges the reasonableness of the first and last connected sample points (Figure 4a). Then, we end the detection if the sample points belong to the obstacle space. If the sample points belong to the self-use space, we select the test point in both directions gradually along the first and last connected sample points and judge the reasonableness of the test point. If the selected test point belongs to the obstacle space, the detection is stopped to discard the path, as shown in Figure 4b. The sample points are connected to each other by collision detection, and finally form a roadmap G(V,E).

### 2.4. Neighbouring Layer Connection Strategy

In the roadmap G(V,E), the threshold distance between road points is an important factor affecting the efficiency of roadmap construction. The path formed by connecting road points in the same sampling layer is not conducive to shorten the global path length. Taking the distribution characteristics of the longitudinal sampling layer into account, we set the connection threshold of the longitudinal sampling spacing $L_{TH}$ to screen the paths that met the threshold conditions and make the connection between road points from the full connection to adjacent sampling layer connection, improving roadmap construction efficiency.

**Figure 5:**
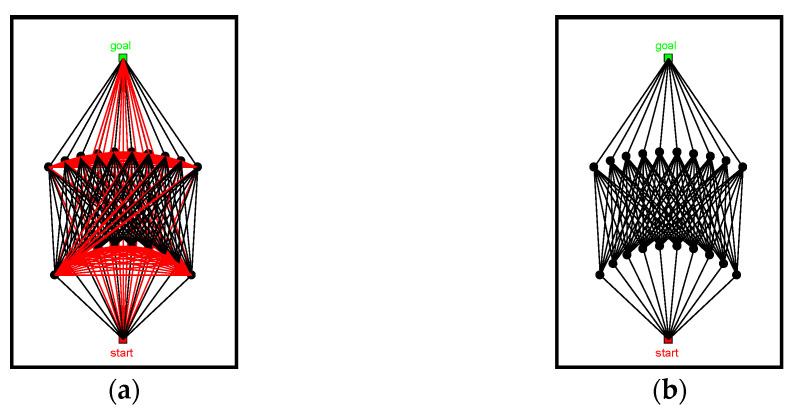
Comparison of road signs: (**a**) full connection and (**b**) neighbouring layer connection.

The sampling points generated based on the pseudo-random sampling strategy (N=20) were selected to obtain the roadmap constructed under the drive of two connection strategies, as shown in Figure 5. Figure 5a shows the wayfinding graph generated by the full connectivity strategy, with the red solid line representing the filtered paths. Figure 5b indicates the wayfinding graph generated by the neighbouring layer connectivity strategy. In terms of time consumption, the composition time using these different connection strategies was 0.906 s and 0.437 s, respectively, and the latter optimized composition efficiency by 48.2%.

## 3. Path Smoothing

In this paper, Bessel curves were chosen to smooth the paths planned by the modified PRM algorithm.

The n order Bessel curve expressions were defined as:

(9) $$B(t)=\sum_{i=0}^{n}P_{i}b_{i,n}(t),\;\left(t\in \left[0,1\right]\right)$$

where $P_{i}$ represents the n+1 control point of the Bessel curve and $b_{i,n}(t)$ represents the Bernstein basis function. The value of this function is shown in Equation (10):

(10) $$b_{i,n}(t)=C_{n}^{i}t^{i}{\left(1-t\right)}^{n-i}=\frac{n\;!}{(n-i)\;!i\;!}t^{i}{\left(1-t\right)}^{n-i}\;,\;i=0,1,2,\ldots ,n$$

In this paper, a 4th order Searle curve was chosen, and the formula is as follows:

(11) $$B(t)={\left(1-t\right)}^{4}P_{0}+4P_{1}{\left(1-t\right)}^{3}t+6P_{2}{\left(1-t\right)}^{2}t^{2}+4P_{3}(1-t)t^{3}+P_{4}t^{4},\;t\in [0,1]\;$$

The curvature of the Bessel curve at any point κ(t) is:

(12) $$\kappa (t)=\frac{\left|B^{\prime }(t)\times B^{\prime \prime }(t)\right|}{{\left|B^{\prime }(t)\right|}^{2}}$$

Assuming that the planning path $path=\left\{P_{n}\right\}$ consists of a series of discrete points (n≥5), the discrete points are used as the control points $P_{i}$ of the Bessel curve, and the curvature of the Bessel curve κ(P) can be obtained according to Equation (12):

(13) $$\kappa (P)=\frac{P_{x}^{\prime }P_{y}^{\prime \prime }-P_{y}^{\prime }P_{x}^{\prime \prime }}{{\left(P_{x}^{\prime }{}^{2}+P_{y}^{\prime }{}^{2}\right)}^{3/2}}$$

The curvature of the Bessel curve at the starting point is κ(0):

(14) $$\kappa (0)=\frac{3\left|\left(P_{1}-P_{0})\times (P_{2}-P_{1}\right)\right|}{4{\left(P_{1}-P_{0}\right)}^{3}}$$

In this specific implementation, the key waypoints of the path searched by the modified PRM algorithm were extracted, discrete control points of the Bessel curve $P_{i}$ were obtained by discretizing the line between key waypoints, and the discrete points were interpolated and fitted by Equation (9) to realize the smoothing of the path.

## 4. Simulation Test and Analysis

To verify the composition and path planning efficiency of the modified PRM algorithm, MATLAB (MATLAB2018b, MathWorks. Inc., Natick, MA, USA) was used to build a simulation experiment platform and a ROS (ROS1.0, Willow Garage. Inc., Menlo Park, CA, USA) experimental platform was used to verify the correctness of the modified PRM algorithm. Our computer configurations included: a Windows 10 operating system, 512 GB hard disk, and 8 GB RAM.

### 4.1. Comparison of Algorithm Composition Efficiency

**Figure 6:**
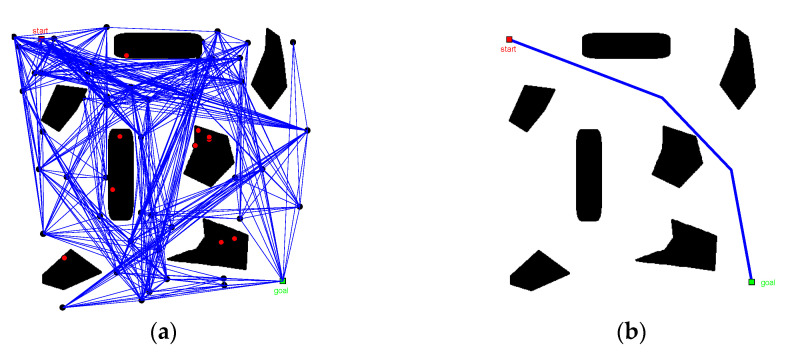
Planning results of the basic PRM algorithm (N=60): (**a**) roadmap and (**b**) planned path.

**Figure 7:**
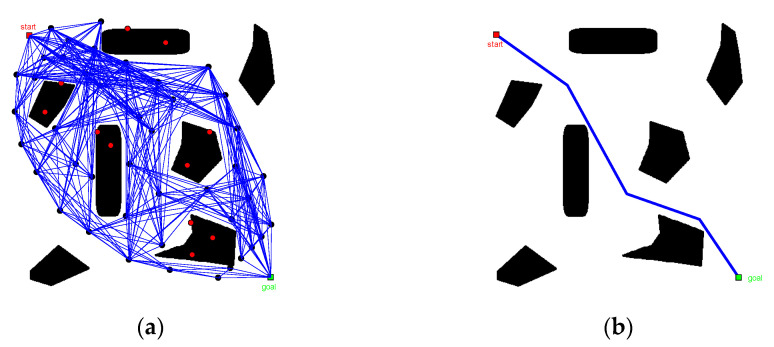
Planning results of the modified PRM algorithm (N=60): (**a**) roadmap and (**b**) planned path.

The planning space of the known map is shown in Figure 6 and Figure 7. The two algorithms kept the same total number N=m×n of sampling points in the sampling phase, where m and n represent the number of horizontal and vertical sampling points of the algorithm, respectively. We focused on the planning path length and roadmap construction time and repeated the test several times (recorded 10 times). The results are shown in Table 1 in mean values.

Taking sampling points N=60 as an example, we analyzed the results of the roadmap construction (Figure 6a and Figure 7a). The sampling points were widely distributed in the PRM algorithm and there were many redundant sampling points. On the other hand, for the roadmap constructed by the modified PRM algorithm (Figure 7a), the location selection of the sampling points had a certain orientation, mainly distributed along the main axis of space, and there were fewer redundant sampling points.

In Figure 6 and Figure 7 and Table 1, it is shown that when the number of sampling points N is 30, the length of the planned path increases by 1.9% and composition time is reduced by 57.8%. When the number of sampling points N is 60, the length of the planned path is reduced by 1.9% and composition time is reduced by 37.1%. When the number of sampling points increase to 90, the length of the planned path is reduced by 5.9% and composition time is reduced 50%. It shows that the changes in path length according to different number N are not consistent. Compared with the PRM algorithm, there is no great advantage in path length for the modified PRM algorithm. However, the modified PRM algorithm showed great advantages in decreasing the construction time of the roadmap; the efficiency of constructing maps was significantly improved.

**Figure 8:**
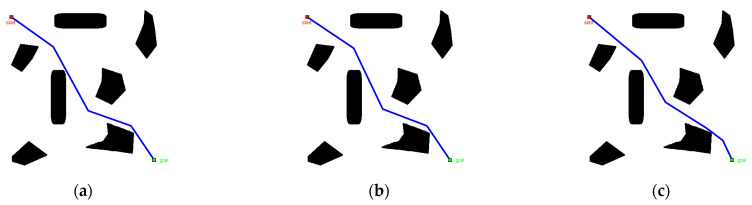
Comparison of planning results of modified PRM algorithm: (**a**) N=30, (**b**) N=60, and (**c**) N=90.

In Figure 8, keeping all other conditions equal, when the number of fold points of the path increased, path smoothness gradually improved as the number of sampling points increased. The overall trend of the path remains unchanged, indicating that the quality of the path solution solved by the modified PRM algorithm is stable.

**Figure 9:**
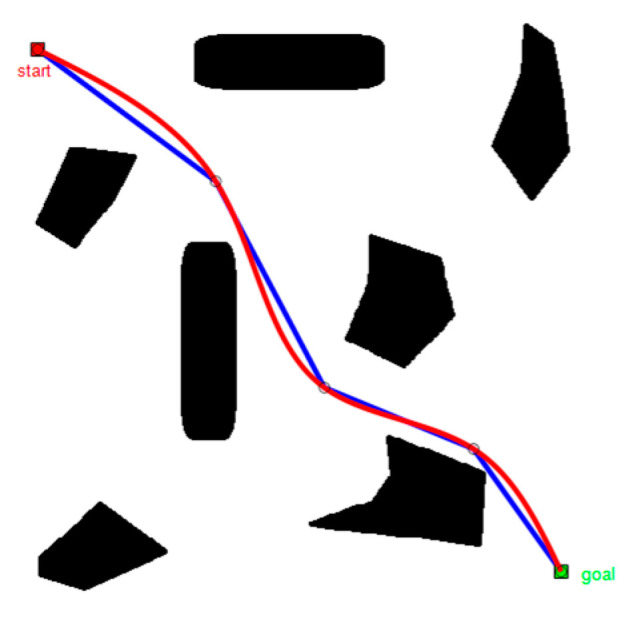
Path smoothing diagram.

To obtain Figure 9, we used the Bessel curve to deal with Figure 8b, the solid blue line indicating the modified PRM algorithm planning path and the black hollow circle logo representing the key road points, used as the Bessel curve control points. The path obtained after the smoothing process (shown by the red line) was more consistent with intelligent vehicle driving road conditions.

### 4.2. Comparison of Path Planning Efficiency

**Figure 10:**
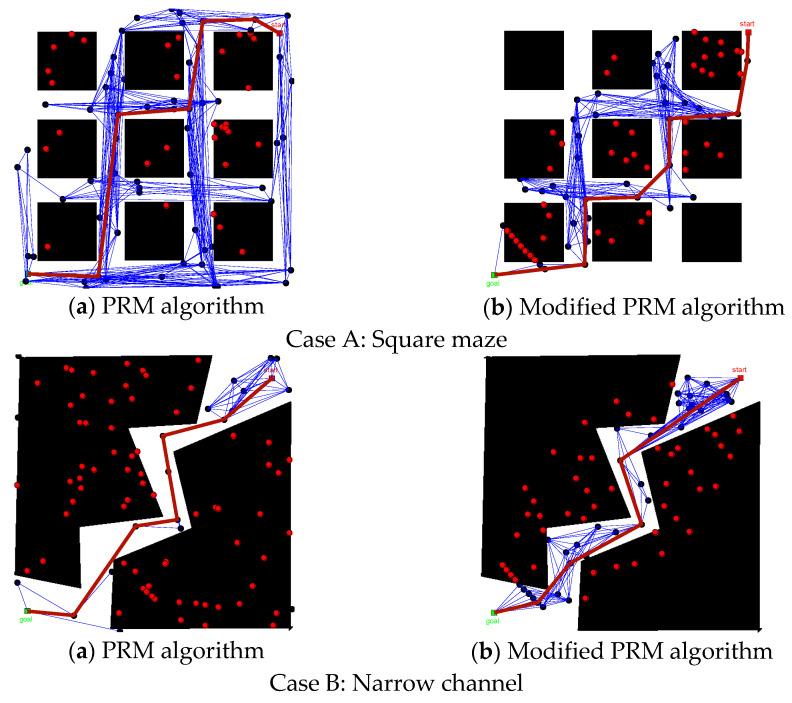
Comparison of algorithm planning results.

**Figure 11:**
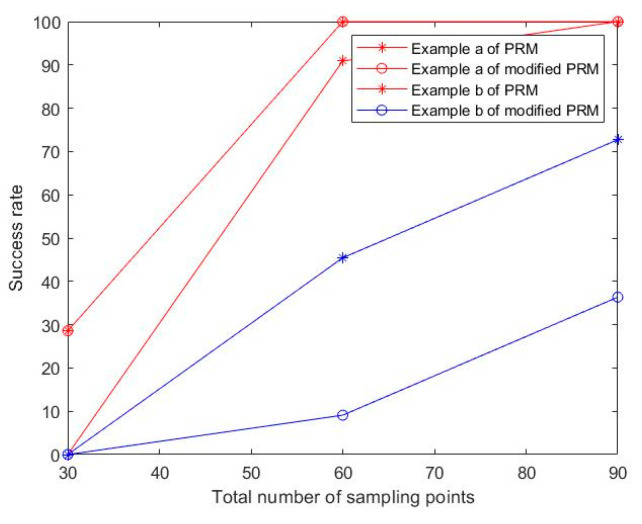
Algorithm success rate comparison.

To verify the path planning efficiency of the modified PRM algorithm, the basic PRM algorithm was used as the comparison algorithm for the case test, where Case A is a square maze and Case B is a narrow channel. The success rate was measured by a ratio of the number of successful path searches to total search number. The results of the case test are shown in Figure 10 and Figure 11 and Table 2.

Referring to Figure 10, in the experiment of Case A, the number of sampling points falling into the obstacle space was comparable in both algorithms, but the sampling points in the self-use space were widely distributed in the PRM algorithm, which caused redundancy. In the modified PRM algorithm, the sampling points were concentrated on both sides of the main axis of the space, which improved the utilization of sampling points. In the experiment of Case B, most of the sampling points in the PRM algorithm fell into the obstacle space, and there were very few sampling points in the self-use space, which affected the quality of the path solution. In the modified PRM algorithm, the sampling points were distributed along the main axis of the space, and the larger number of sampling points in the self-use space provided the possibility of seeking a better path solution.

In Table 2 and Figure 11, for Case A, the modified PRM algorithm could not successfully plan the path when the number of sampling points was low (N=30). When the number of sampling points increased to 60 (N=60), the differences between the two algorithms in path length, running time, and success rate were not obvious. When the number of sampling points increased to 90 (N=90), the modified PRM algorithm was better than the basic PRM algorithm in path length and running time. For Case B, when the number of sampling points was low (N=30), both algorithms could not successfully plan the path, and as the number of sampling points increased, the modified PRM algorithm had a higher success rate in path planning and the quality of the path solution was more reliable.

### 4.3. ROS Simulation Test

**Figure 12:**
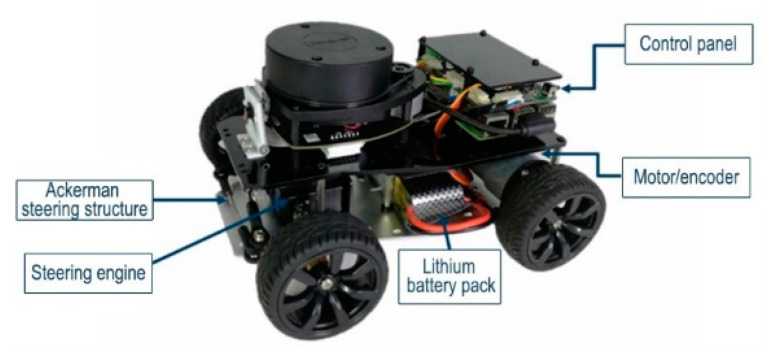
ROS car composition.

In order to further verify the implementability of the modified PRM algorithm, simulation tests were designed, based on the ROS experimental platform. The composition of the ROS trolley is shown in Figure 12.

**Figure 13:**
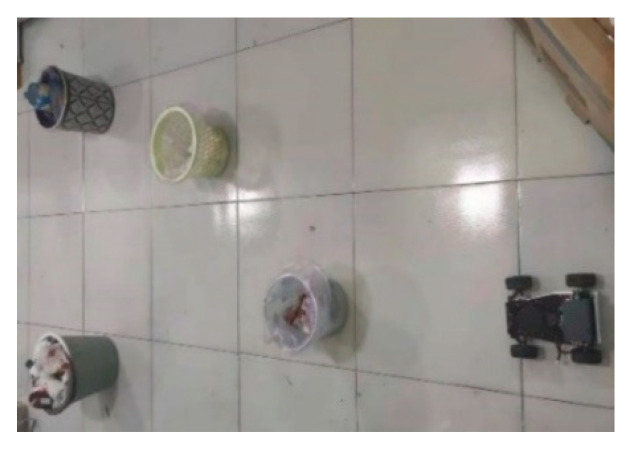
Field map.

**Figure 14:**
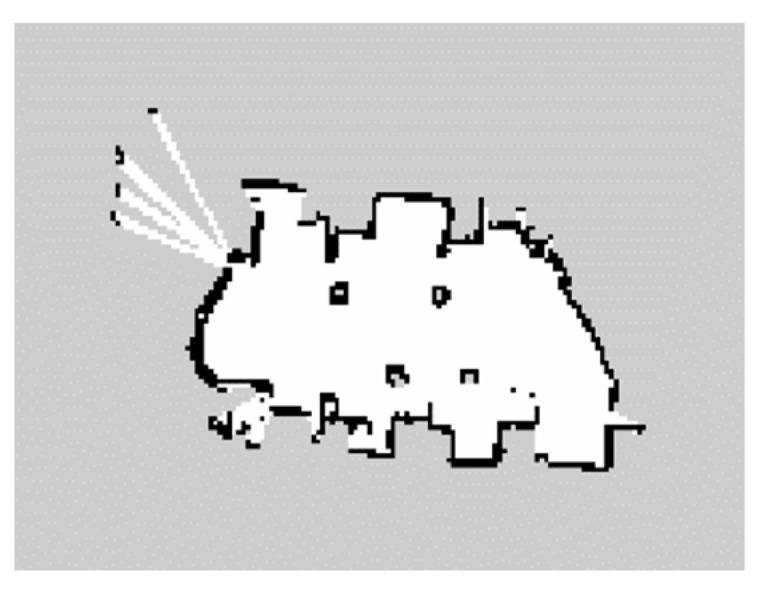
SLAM map.

**Figure 15:**
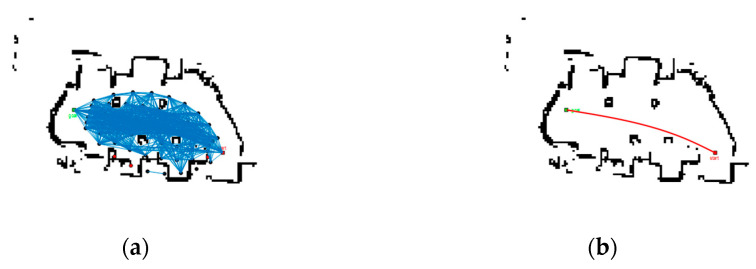
Path planning: (**a**) wayfinding map and (**b**) planning path.

We mainly addressed the path planning problem of smart vehicles in a two-dimensional environment, using the function package provided by the ROS experimental platform to implement the LIDAR map building function. The test site is shown in Figure 13, and the SLAM map building effect is shown in Figure 14. Based on this environmental map, we defined the localization result of ROS itself as the starting point and specified the target point. The modified PRM algorithm was executed and the path planning results are shown in Figure 15.

From the simulation results, a road map was established in the SLAM map by the modified PRM algorithm. Meanwhile, the modified PRM algorithm planned a path successfully connecting the starting and target point, verifying the feasibility of the modified PRM algorithm.

## 5. Conclusions

In order to improve the overall quality of the PRM algorithm in path planning, a pseudo-random sampling method based on uniform sampling was designed to optimize the quality of sampling point generation. Random increments were introduced to adjust the fluctuation range of sampling points to effectively avoid the obstacle space. Due to the disadvantage of a low rate of roadmap construction, a two-way incremental collision detection strategy was used to set the connection threshold between road points to reduce the number of collision detection calls. Finally, the correctness of the modified PRM algorithm was verified and analyzed using MATLAB and ROS test platforms. The test results showed that the modified PRM algorithm has obvious advantages in enhancing the stability of the roadmap, shortening the length of the planned path, and improving the search rate of the algorithm. However, the majority of current algorithms, including the modified PRM algorithm, are model-driven, and face many limitations. These algorithms need to be further researched. Data-driven and cloud-network fusion technologies could be added to these algorithms to achieve better path planning and obstacle avoidance in smart vehicles.

## Figures and Tables

**Table 1:** The results of algorithm comparison.

Algorithm Name	Number of Sampling Points N	Path Length/m	Composition Time/s
PRM algorithm	30	582.1	0.958
	60	602.6	3.269
	90	615.4	10.393
Modified PRM algorithm	30	593.3	0.404
	60	590.6	2.056
	90	578.7	5.196

**Table 2:** Comparison results of algorithm efficiency.

Algorithm Case	Sampling Points N	Basic PRM Algorithm			Modified PRM Algorithm		
		Path Length/m	Running Time/s	Success Rate/%	Path Length/m	Running Time/s	Success Rate/%
**A**	30	883.14	0.23	28.57	\	\	0
	60	869.63	0.89	100	839	0.62	90.91
	90	861.86	2.18	100	819.91	1.12	100
**B**	30	\	\	0	\	\	0
	60	812.1	0.28	9.09	735.51	0.29	45.45
	90	734.53	0.43	36.36	729.45	0.73	72.73

## Data Availability

The study did not report any data.
